# Polyisoprenylated methylated protein methyl esterase as a putative drug target for androgen-insensitive prostate cancer

**DOI:** 10.3332/ecancer.2014.459

**Published:** 2014-08-28

**Authors:** Rosemary A Poku, Felix Amissah, Randolph Duverna, Byron J Aguilar, Gebre-Egziabher Kiros, Nazarius S Lamango

**Affiliations:** College of Pharmacy and Pharmaceutical Sciences, Florida A&M University, Tallahassee, Florida 32307, USA

**Keywords:** carboxylesterase 1, esterase, isoprenylation, PMPMEase, polyisoprenylation, prenylation, protein methylation

## Abstract

Prostate cancer (CaP) is the most frequently diagnosed cancer in US men, with an estimated 236,590 new cases and 29,720 deaths in 2013. There exists the need to identify biomarkers/therapeutic targets for the early/companion diagnosis and development of novel therapies against the recalcitrant disease. Mutation and overexpression-induced abnormal activities of polyisoprenylated proteins have been implicated in CaP. Polyisoprenylated methylated protein methyl esterase (PMPMEase) catalyses the only reversible and terminal reaction of the polyisoprenylation pathway and may promote the effects of G proteins on cell viability. In this review, the potential role of PMPMEase to serve as a new drug target for androgen-insensitive CaP was determined. Specific PMPMEase activities were found to be 3.5- and 4.5-fold higher in androgen-sensitive 22Rv1 and androgen-dependent LNCaP and 1.5- and 9.8-fold higher in castration-resistant DU 145 and PC-3 CaP cells compared to normal WPE1-NA22 prostate cells. The PMPMEase inhibitor, L-28, induced apoptosis with EC_50_ values ranging from 1.8 to 4.6 μM. The PMPMEase activity in the cells following treatment with L-28 followed a similar profile, with IC_50_ ranging from 2.3 to 130 μM. L-28 disrupted F-actin filament organisation at 5 μM and inhibited cell migration 4-fold at 2 μM. Analysis of a CaP tissue microarray for PMPMEase expression revealed intermediate, strong, and very strong staining in 94.5% of the 92 adenocarcinoma cases compared to trace and weak staining in the normal and normal-adjacent tissue controls. The data are an indication that effective targeting of PMPMEase through the development of more potent agents may lead to the successful treatment of metastatic CaP.

## Introduction

Castration-resistant prostate cancer (CRPC) continues to be a major challenge in the therapeutic management of prostate cancer (CaP). There were over 236,590 estimated new cases and 29,720 deaths from the disease in 2013 in the United States alone [1]. Androgen deprivation has been the first-line therapy for metastatic CaP for many years [2]. This is accomplished by surgical or chemical castration [3]. Although most human CaP initially depend on androgens for growth, they eventually become androgen-insensitive at varying intervals following androgen ablation [2]. A 2–3-year remission period is usually achieved, after which virtually all patients’ disease progress to an androgen-independent state that results in death within 16–18 months [2]. This CRPC is marked by rising serum prostate-specific antigen (PSA), increasing the tumour size and new metastatic capability [3]. The emergence of hormone resistance has been the issue of concern in the management CRPC since CaP is not fatal until it becomes castration-resistant and metastatic. Unfortunately, no effective alternative treatments have been found in the hormone-refractory tumours [4]. The need for novel targets for the development of effective therapies is therefore imperative.

The normal development, growth, and survival of the prostate epithelium are regulated by the secretion of androgens and growth factors, such as epidermal growth factor (EGF), transforming growth factor alpha (TGFα), keratinocyte growth factor (KGF), basic fibroblast growth factor (bFGF), and insulin-like growth factor 1 (IGF-1), in the stroma by paracrine glands. Similar regulatory interactions occur between androgens and growth factors in CaP [4]. The androgen receptor (AR) signalling axis plays a critical role in the development, function, and homeostasis of the prostate. As a result, the initiation and progression of CaP depend heavily on the AR. AR regulates gene transcription through its nuclear translocation, binding to androgen response elements on target genes and recruitment of or cross talk with transcription factors [5]. This explains why androgen-deprivation therapy remains the standard care for treatment for advanced CaP [6]. Studies have shown that when CaP cells are stimulated by mitogenic factors, they lose their dependence on androgens for survival and growth. Overexpression of EGF, TGF-α, KGF, βFGF, IGF-I and related ligands has also been reported in advanced CaP [7]. The epidermal growth factor receptor (EGFR or RTK) is overexpressed in 41.4%, 75.9%, and 100% of patients who were treated by radical prostatectomy, LHRH/antiandrogens/radical prostatectomy, and those who had developed CRPC, respectively [8]. Inhibition of EGFR signalling causes significant reduction of CaP cell growth [9]. However, the androgen-dependent CaP cells have been shown to develop resistance to anti-EGFR drugs by synthesising more EGFR as well as expressing HER2, HER3, and HER4 receptors [10]. In other studies, CRPC cells did not show sensitivity to dual EGFR-HER2 inhibition [11].

Ras is a key downstream effector for the growth factor receptors whose signalling is amplified in advanced CaP [4]. Although Ras is rarely mutated in the CaP of western patients, somatic Ras mutations have been detected in 24% of Japanese CaP patients [12]. Ras is, however, a common signalling mediator protein for the growth factors and their receptors and is therefore an appropriate target for therapeutic intervention [4]. Furthermore, changes that make Ras hyperactive result in tumours that are resistant to anti-EGFR therapies given that Ras appears downstream of EGFR [13]. Therefore, targeting hyperactive Ras might control not only tumours spurred by this aberration but also those that are promoted by upstream events such as overexpressed and/or mutated hyperactive EGFR. Tumours with mutated Ras neither show progression-free survival nor benefit from anti-EGFR tyrosine kinase inhibitors (TKIs) or monoclonal antibodies (MAbs) [14, 15]. As a result, the patients with hyperactive Ras have poorer prognoses and worse clinical outcomes than patients with hyperactive EGFR [15, 16]. Furthermore, only a fraction of the patients with wild-type Ras respond to anti-EGFR therapy, to which they soon develop resistance [17]. Even among patients with hyperactive EGFR, a large proportion is resistant to anti-EGFR therapies [18]. Consequently, there is widespread agreement that new approaches for early diagnosis and treatment are needed for patients who do not benefit from or develop resistance to anti-EGFR drugs [16, 19]. 

Ras comprises a family of small guanine nucleotide-binding proteins that are polyisoprenylated to enable them to perform their cellular regulatory roles in growth, survival, and differentiation [20]. Polyisoprenylation is an essential secondary modification of a wide range of proteins in eukaryotic cells. These proteins have been widely reported to contribute to diseases due to mutations or improper metabolism [21]. Proteins that are polyisoprenylated undergo reversible methylation catalysed by polyisoprenylated protein methyl transferase (PPMTase) and polyisoprenylated methylated protein methyl esterase (PMPMEase) [22]. These enzymes appear to play intricate roles in ensuring that appropriate pools of methylated and demethylated polyisoprenylated proteins are available for normal physiological function.

In this study, we hypothesised that PMPMEase may be hyperactive in and contribute to CaP progression. As such, PMPMEase would be a suitable target for therapeutic management given our previous observations that its inhibition induces cancer cell death [23–26]. In the current study, we observed intermediate to very strong expression of PMPMEase in 94.5% of 92 adenocarcinoma cases relative to normal or normal-adjacent tissues. Its overexpression results in an almost 10-fold hyperactivity in castration-resistant CaP cells that become apoptotic upon treatment with a specific farnesylated irreversible PMPMEase inhibitor. PMPMEase inhibition in these cells also disrupts F-actin organisation, significantly inhibiting the migration of the CaP cells. Taken together, this study suggests that PMPMEase inhibition holds promise in the therapeutic management of androgen-insensitive metastatic CaP.

## Materials and methods

### Cell culture

Normal human prostatic epithelial WPE1-NA22 cells, castration-resistant human CaP PC3 and DU145 cells, androgen-dependent human CaP LNCaP, and androgen-sensitive 22Rv1 cells were purchased from American Type Culture Collection (Manassas, VA, USA) who also authenticated and certified them. WPE1-NA22 cells were cultured in keratinocyte serum-free media supplemented with 0.05 mg/ml BPE and 5 ng/ml EGF (Invitrogen, Carlsbad, CA, USA). PC-3 cells were maintained in F12K medium (Invitrogen), DU145 cells were cultured in Eagle’s Minimum Essential Medium (Invitrogen) while LNCaP and 22Rv1 were maintained in RPMI 1640 medium (Invitrogen). Except media for the normal prostate cells, all other media were supplemented with 10% fetal bovine serum (FBS), 100 U/ml penicillin, and 100 mg/ml streptomycin (Invitrogen). The cells were incubated at 37 °C in a humidified atmosphere of 95% air and 5% CO_2_. N-(4-nitrobenzoyl)-S-trans, trans-farnesyl-L-cysteine methyl ester (RD-PNB) substrate was synthesised in our laboratory, as previously described [22]. 

### Assay for PMPMEase

Cells were cultured in 175 cm^2^ vented culture flasks to about 80% confluence, washed with PBS, lysed with 0.1 % Triton-X 100 in 100 mM Tris–HCl, pH 7.4 containing 1 mM EDTA. The protein concentrations in the lysates were determined using the bicinchoninic acid assay (BCA) method. Aliquots of the resulting lysates were incubated with 1 mM RD-PNB in a total reaction volume of 100 μL at 37°C for 3 h. The effect of 2-*trans, trans*-farnesylthioethanesulfonyl fluoride (L-28) on PMPMEase activity in the lysates was determined by conducting the enzyme assay in the presence of varying concentrations of L-28. All reactions were stopped by the addition of methanol followed by HPLC analysis, as previously described [22]. 

### PMPMEase levels in prostate cells

Cells were washed with PBS and lysed in modified RIPA buffer (150 mM NaCl, 50 mM Tris-HCl, 50 mM NaF, 5 mM EDTA, 0.5% w/v sodium deoxycholate, and 1% Triton X-100) containing phosphatase and protease inhibitors. The protein concentrations of the lysates were determined using the BCA method. Aliquots of the lysates containing 50 μg of protein were mixed with SDS-PAGE sample buffer and subjected to SDS-PAGE followed by western blotting using rabbit polyclonal antibody directed against human carboxylesterase 1 and alkaline phosphatase-conjugated goat anti-rabbit secondary antibody (Santa Cruz Biotechnology, CA, USA). Immunoreactivity was detected using 5-Bromo-4-chloro-3-indolyl phosphate/Nitro blue tetrazolium BCIP/NBT (Sigma, St Louis, MO, USA).

### Cell viability assay

PC-3, DU145, LNCaP, and 22Rv1 cell lines were seeded at a density of 2 × 10^4^ per well, while normal prostate cells (WPE1-NA22) were seeded at 5 × 10^4^ cells per well in 96-well tissue culture plates. Cells were incubated overnight at 37 °C in 5% CO_2_/95% humidified air. The cells were then treated with varying concentrations of L-28 (1-200 μM in 1 μl of acetone) for 24, 48, and 72 h. CellTitre-Blue Cell Viability Assay kit (CTB) (Promega, Madison, WI, USA) was used to determine the cell viability as previously described, and the results were expressed as percentages of the fluorescence of the untreated control cells [25]. 

To determine the mode of cell death, CaP cells were seeded at a density of 2 × 10^5^ per well in 24-well culture plates overnight. The cells were treated with L-28 for 24 and 48 h, after which they were stained with acridine orange (AO)/ethidium bromide (EB), and the fluorescence images were taken as previously described [26]

### Effect of PMPMEase inhibition on cell migration and F-actin organisation

PC-3 cells were seeded into 12-well plates for 24 h. The cells were then serum-starved for an additional 24 h, after which a scratch was carefully made in the monolayer with a 10 μl pipette. HBSS was used to wash off the debris and followed by the addition of complete media. The cells were then treated with solvent (acetone), 1 and 2 μM L-28. Images were taken at 0, 6, 12, and 24 h at 100× magnification.

PC-3 cells were cultured in 24-well plates at a density of 2 × 10^5^ per well and treated with L-28 (1–50 μM for 48 h). The cells were washed twice with PBS and fixed with 3.7% formaldehyde in PBS for 10 min. The cells were then blocked with 1% BSA in PBS for 20 min before incubating with fluorescein-tagged phalloidin (Biotium, CA, USA) for 1 h at room temperature. The cells were then washed in PBS and stained with 4,6-diamidino-2-phenylindole (DAPI) at 0.1μg/ml in to visualize the nuclei. The fluorescent images were taken as previously described [26]. 

### Effect of PMPMEase inhibition on the expression of cancer-related genes

LNCaP, 22Rv1, PC-3, and DU 145 were plated in 24-well plates at a density of 1 × 10^5^ and incubated for 24 h. These were then treated with solvent (acetone), L-28 (2 or 5 μM) for 48 h. The cells were then harvested and counted, and 10,000 cells from each treatment group were lysed in 5 μl of RLT buffer. The mRNA levels were analysed using the NanoString nCounter GX Human Cancer References Kit for profiling cancer-related genes (NanoString Technologies, Seattle, WA, USA) at the Oncogenomics Core Facility, Sylvester Comprehensive Cancer Center of the University of Miami Health System. The raw gene counts obtained from the NanoString were analysed using the nSolver software (NanoString Technologies) as described by the manufacturer.

### PMPMEase expression in CaP

The expression of PMPMEase in CaP tissues was studied using immunohistochemical analysis of CaP and normal-adjacent tissue microarray (TMAs) composed of a total of 208 cores from 114 cases. The human TMAs used in the studies were supplied by, and the immunohistochemistry conducted at US Biomax (Rockville, MD, USA). These were probed and analysed for PMPMEase (human carboxylesterase 1, hCE1) as described previously [24].

### Statistical analysis

All results were expressed as the means ± SEM. Nonlinear regression plots were generated using GraphPad Prism analysis software (San Diego, CA, USA). From the graphs, the concentrations that inhibited 50% of the activity (IC_50_) were obtained. The TMA data were analysed by one-way ANOVA using SAS 9.2 Software (SAS Institute, Cary, NC, USA). Statistical differences in mean intensities between normal and cancer tissues were determined using Bonferroni multiple comparisons. All tests were two sided, and p-values < 0.05 were considered significant.

## Results

### PMPMEase protein and enzymatic activity levels are elevated in CaP cells

**Figure 1.**
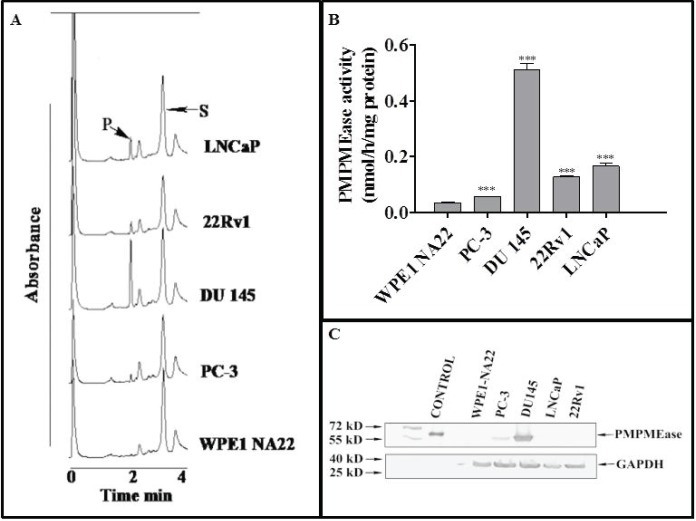
PMPMEase is hyperactive in prostate cancer cells. Cell lysates (1.3–2.8 mg of protein) were incubated with RD-PNB substrate (1 mM) at 37°C and the reaction mixtures were analysed by HPLC as described in the methods. Panel A shows HPLC chromatograms for the analysis of samples from the different prostate cells for PMPMEase hydrolysis of the substrate, while Panel B shows the computed specific PMPMEase activities in the cell lysates. Panel C shows the relative PMPMEase protein levels determined by Western blotting when compared to the pure PMPMEase (3.3 µg, positive control) and normal prostate cells by paired *t*-test. ***p < 0.0001.

Incubation of the specific PMPMEase substrate, RD-PNB, with lysates from the respective cell lines resulted in the hydrolysis of the substrate to the product as determined by HPLC analysis with UV detection (Figure 1A). The specific PMPMEase activities followed the same pattern as the product peak areas observed in the chromatograms (Figure 1B). The specific PMPMEase activities were 3.5- and 4.5-fold higher in androgen-sensitive 22Rv1 and androgen-dependent LNCaP cells and 1.5- and 9.8-fold higher in castration-resistant PC-3 and DU 145 CaP cells compared to normal WPE1-NA22 prostate cells. When the various CaP cell lines were lysed and aliquots containing identical amounts of protein were analysed by western blotting, PMPMEase was observed to be highly expressed in the castration resistant DU 145 and PC-3 cells compared to the normal prostate, androgen-dependent LNCaP and androgen-sensitive 22Rv1 cells (Figure 1C).

### Inhibition of PMPMEase activity in prostate cells induces apoptosis

**Figure 2.**
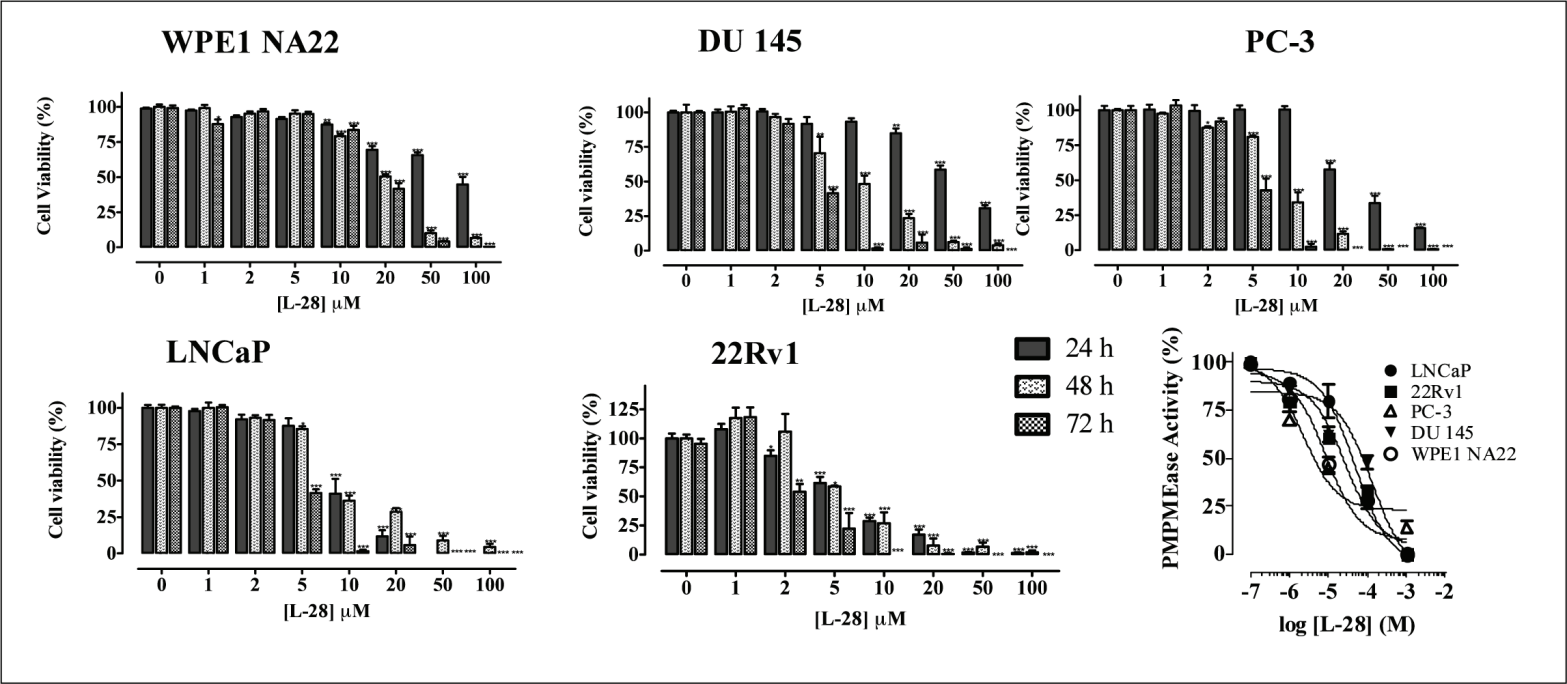
L-28 induces apoptosis in prostate cancer cell lines. Cells were plated in 96-wells at a density of 2×10^4^ as described in the methods. At 24, 48 and 72 h of treatment with varying concentrations of L-28, the cell viabilities were measured by fluorescence using the resazurin reduction assay. The bottom right panel shows the inhibition of PMPMEase activity in the different cell lysates by L-28. Each point represents the mean ± SEM of 4 determinations. *p < 0.01, **p < 0.001, and ***p < 0.0001 when compared to the normal prostate cells by paired t-test.

Studies with polyunsaturated fatty acids [25] and sulfonyl fluorides [23] revealed that PMPMEase hyperactivity might be involved in cancer cell viability and proliferation. In the present study, both androgen-dependent and independent CaP cells were treated with the specific PMPMEase inhibitor, L-28, and analysed for its effects on cell viability and apoptosis. As shown in Figure 2, L-28 suppressed the viability of the CaP cells at low micromolar EC_50_ values (Table 1). On the contrary, the EC_50_ values for the normal WPE1-NA22 prostate cells were 8- and 21-fold higher than those for the cancer cells after 72 h of exposure to L-28.

When lysates from the respective cell lines were incubated with the substrate in the presence of varying concentrations of L-28, a concentration-dependent inhibition of the PMPMEase enzymatic activity with IC_50_ values ranging from 3 (PC-3) to 130 μM (DU 145) were observed (Figure 2).

**Figure 3.**
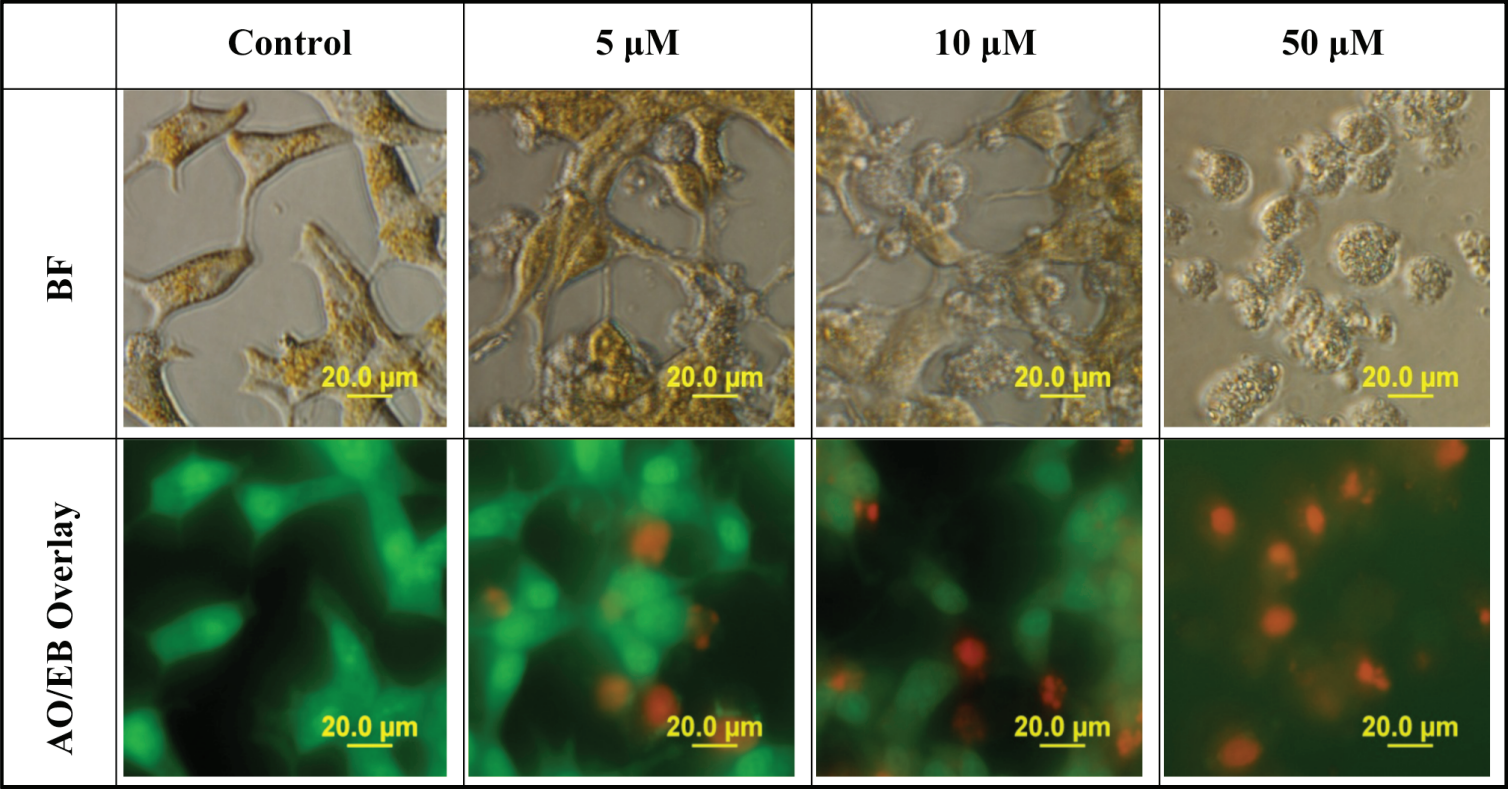
L-28 induces cell death by apoptosis. Cells exposed to 0–50 μM of L-28 for 48 h in serum-free medium were treated with AO/EB (10 μg/ml), as described in the methods. The images were then taken with an Olympus DP70 Camera fluorescent microscope, as described in the methods.

When determining the mode of cell death, AO easily permeates all cells, staining their nuclei green, while EB permeates only the cells with lost cytoplasmic membrane integrity and stains their nuclei red. An overlay of AO/EB shows the mode of cell death. Figure 3 reveals a decline in the number of cells stained with the green colour of AO and a gradual increase in the number of cells stained with the orange colour of EB as concentrations of L-28 increase in the treated LNCaP cells. Following treatment with L-28, the nuclei of the viable LNCaP cells were stained green, while condensed apoptotic nuclei in the treated groups were stained orange and/or red (Figure 3). When the cells were exposed to 50 μM of L-28, nuclear fragments depicting apoptotic bodies were observed (Figure 3).

### PMPMEase inhibition alters cell morphology, disrupts F-actin organisation and cell mobility

**Figure 4.**
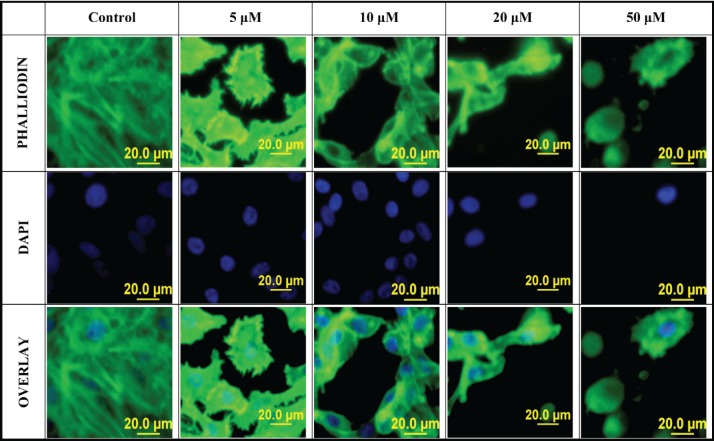
Disruption of F-actin organisation in PC-3 cells by L-28: PC-3 cells were treated with L-28 (0–50 μM) for 48 h, fixed with 3.7% paraformaldehyde and permeabilised with 0.1% Triton X-100. These were then probed with fluorescein-conjugated phalloidin and DAPI, as described in methods. The localisation of F-actin filaments and nuclei are depicted by the green and blue colours, respectively. The fluorescent images were captured using a fluorescent microscope equipped with an Olympus DP70 camera.

When PC-3 cells were treated with varying concentrations of L-28, cell morphology was significantly altered, displaying a more retracted morphology compared to the more expansive control untreated cells. This was accompanied by the collapse of F-actin filaments as observed using the phalloidin staining after 48-h exposure to L-28 (Figure 4). The integrity of F-actin stress fibres was maintained in the control untreated PC-3 cells.

**Figure 5.**
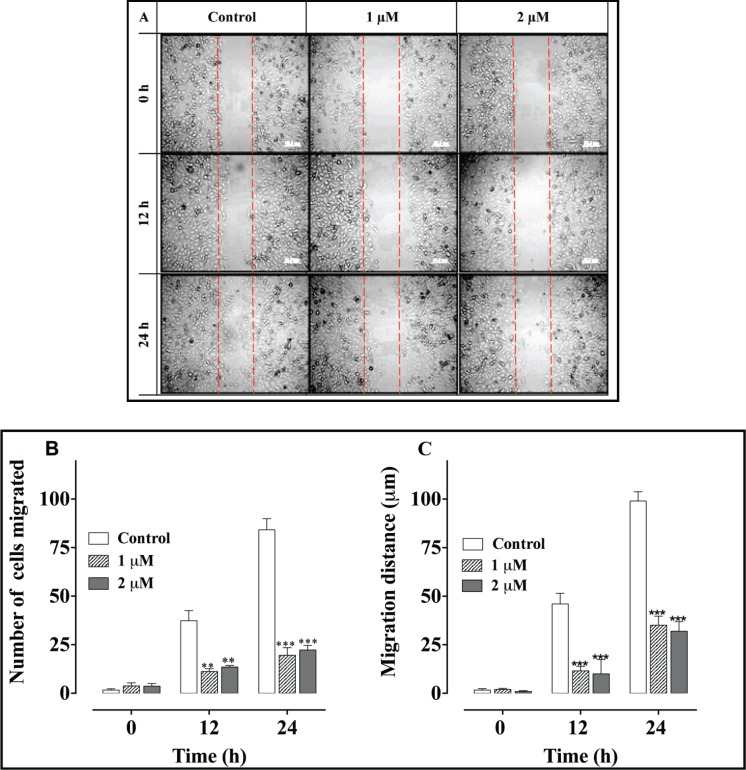
L-28 inhibits PC-3 cell migration: a monolayer of PC-3 cells was scratched with a 10 μl pipette tip and treated with L-28 for 24 h, as described in the methods. Panel A shows images of the cells captured at 0, 12, and 24 h post-scratch and treatment with L-28. Panels B and C show the number of cells that migrated into the scratch area and the migratory distance plotted against time. **p < 0.01 and ***p < 0.001.

A scratch (wound-healing) cell migration analysis of PC-3 cells exposed to 1 and 2 μM concentrations of L-28 revealed significant inhibition of cell migration compared to the controls (Figure 5). The average numbers of migratory cells were 2.7- and 3-fold lower than controls after 12 h and 3.7- and 4-fold lower than in the controls after 24 h exposure to L-28, respectively (Figure 5B). As shown in  Figure 5C, the migratory distance followed a similar pattern, with L-28 significantly inhibiting the motility of the PC-3 cells.

### PMPMEase inhibition alters the expression of cancer-related genes

**Figure 6.**
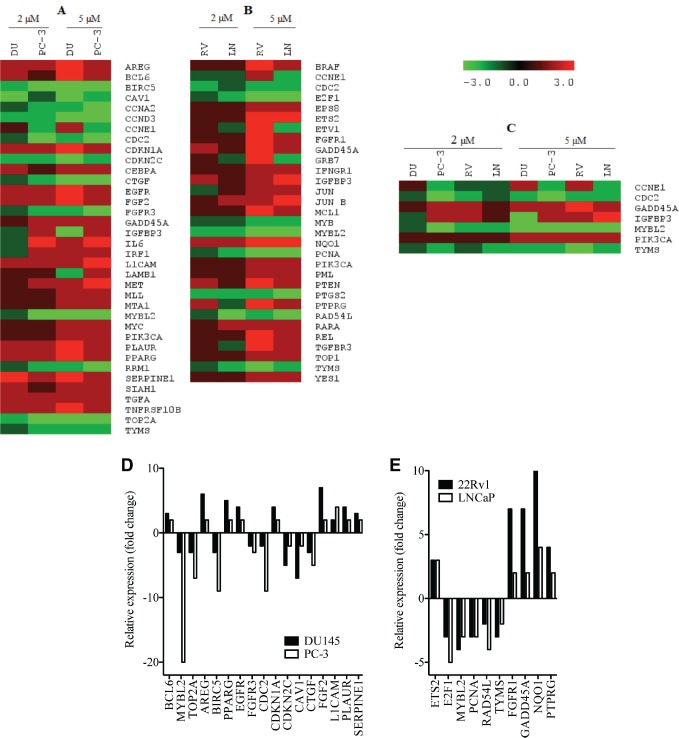
L-28 alters the expression of cancer-related genes in prostate cancer cell lines: the CaP cells were treated either with 0 (controls), 2 or 5 μM L-28 for 48 h. The cells were lysed and analysed for the respective mRNA levels using the NanoString nCounter system, which uses highly specific molecular barcodes to count the number of each type of target mRNA. Panel A: altered gene expression in androgen- independent DU 145 and PC-3. Panel B: altered gene expression in androgen-dependent 22Rv1 and LNCaP cells. Panel C: Altered gene expression in all four CaP cell lines used. Panels D and E show a graphical representation of the most affected genes.

In order to better understand the molecular basis for the observed effects of PMPMEase inhibition on the cancer cells, the four CaP cell lines were treated with 0, 2, and 5 μM concentrations of L-28 for 48 h to minimize possible cell death. L-28 (2 and 5 μM) altered the expression of several important genes in the four CaP cell lines studied. Considering a 2-fold increase or decrease in gene expression as significant, 50, 104, 54, and 98 genes were affected in LNCaP, 22Rv1, PC-3, and DU 145 cell lines, respectively. The altered expression of 36 genes was common to the two castration-resistant PC-3 and DU 145 cells (Figure 6A), while the expression of 32 genes was common to the androgen-sensitive 22Rv1 and androgen-dependent LNCaP cells (Figure 6B). Also, the expression of the genes that encode for cyclin E1 *(CCNE1)*, cyclin-dependant kinase 1 *(CDC2)*, *GADD45A, TYMS, MYBL2, IGFBP3* and *PIK3CA* was altered across all four CaP cell lines (Figure 6C). The products of these genes play significant roles in cell cycle progression, arrest and apoptosis, DNA synthesis and repair, gene transcription and metastasis. These genes were significantly suppressed in the castration-resistant cell lines including TOP2A, which encodes for Topoisomerase IIα, a critical nuclear enzyme that induces changes in the DNA geometry during its untangling and movement. Cyclin D3 *(CCND3)*, cyclin A2 *(CCNA2)* as well as antiapoptotic genes, such as *BIRC5* and *CTGF* that stimulate cell proliferation, adhesion and metastasis also showed a decreased expression. The expression of cyclin-dependant kinase inhibitor, *CDKNIA* (p21), which induces cell cycle arrest, increased by up to 4-fold in DU 145 cells following treatment with the PMPMEase inhibitor. Genes that code for EGFR and its ligands, AREG as well as growth factors, such as FGF2 that utilize the MAP Kinase pathway, were upregulated in the castration resistant cell lines (Supplemental Table 1).

The pro-apoptotic gene, *PTEN*, and the tumour suppressor gene, *PML*, were upregulated in the androgen-dependent cell lines following treatment. Also, the expression of the important early response transcription factor genes *E2F1* that aids in ushering cells in G0 phase back into the cell cycle was significantly suppressed. The expression of *NQO1* that protects cells against oxidative stress was stimulated by the treatments. Increased expression of genes that code for such growth factors and their receptors as EGFR, EPS8, and FGFR1 in the EGF/MAP kinase pathway was observed with L-28 treatment (Supplemental Table 2).

### PMPMEase hyperactive in CaP tissues

Analysis of a CaP TMA for PMPMEase expression revealed intermediate, strong, and very strong staining in 94.5% of the 92-adenocarcinoma cases compared to only trace and weak staining in the normal and normal-adjacent tissue controls. The clinicopathological features of patients whose tissues were used in the TMAs are shown in Supplemental Table 4. This included 92 adenocarcinoma, two transitional cell carcinoma, eight normal, and 12 normal-adjacent tissue (NAT) specimens, with patients’ ages ranging from 19 to 81 years. Gleason grades ranged from 1 to 5, with most tumours falling within a grade range of 3–5. The Gleason scores ranged from 2+2 = 4 to 5+5 = 10, with a majority of tumours having scores of 5, 6, 8, and 10.

**Figure 7.**
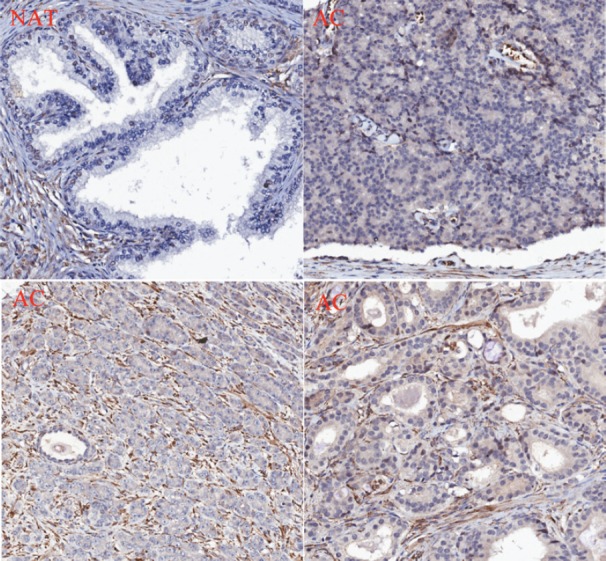
PMPMEase is overexpressed in prostate cancer. A prostate cancer TMA was probed by immunohistochemical analysis for the relative expression of PMPMEase as described in the methods. Intense staining was observed in the prostate adenocarcinoma (AC) compared to the normal-adjacent tissue (NAT).

PMPMEase staining was observed in the perinuclear endoplasmic membrane space and the cytoplasm of stromal prostatic tissues, while in the tumour cells, intense staining was observed both in the glandular and stromal cells (Figure 7). It is worth noting that normal and NATs revealed distinct demarcations between glandular and stromal prostatic cells, whereas the two portions are merged in the tumours (Figure 7). A significant difference in PMPMEase staining was observed in normal, NAT (*p* < 0.0094), and tumour tissues (*p* < 0.0001); a higher Gleason grade or score correlated with intermediate, strong, and very strong staining as shown in Supplemental Table 3. The mean staining intensity score for normal prostatic tissues was 163 ± 38, with 75% showing trace to weak staining, while that of prostate adenocarcinoma tissues was 348 ± 8.3. These results indicate that PMPMEase is overexpressed in CaP tissues compared to normal prostate tissues.

## Discussion

PMPMEase was first isolated in our laboratory using a specific polyisoprenylated ester substrate to assay chromatographic fractions. This resulted in a single esterase that was identified as the porcine ortholog of human carboxylesterase 1 (hCE1) [28]. We later found that chemopreventive food-derived substances that inhibit the enzyme had profound inhibitory effects on the viability of cancer cells [24, 25]. These findings, in addition to the roles that polyisoprenylated proteins play in cancer progression led us to hypothesise that PMPMEase may be hyperactive in cancers. This suspicion was recently confirmed when PMPMEase was found to be overexpressed in 86.6% of colorectal cancer cases [24]. In recognition of this and the ubiquitous nature of polyisoprenylated proteins, we examined the expression of PMPMEase in CaP tissues and cell lines. The observation that PMPMEase is hyperactive and overexpressed in CaP cell lines and that 87% of CaP cases is congruent with the effects of PMPMEase inhibition on cell viability not only on the CaP cells, but also in the lungs [25, 51] and colorectal cancer cell lines [24]. These results have a direct bearing on such aspects of cancer biology as proliferation and metastasis, given the biological functions of PMPMEase putative protein substrates on cell viability and migration. For example, it was recently reported that siRNA directed against hCE1 (PMPMEase), but not CES2, inhibited RhoA demethylation and altered the morphology of breast cancer cells [29]. Furthermore, an overexpression of polyisoprenylated protein methyl transferase (PPMTase/icmt) in the same cell line induced the opposite effects both on RhoA metabolism and cell morphology [29]. These reports are consistent with the morphological changes observed in cells in which PMPMEase moderately inhibited [23, 25, 26, 51]. The current study indicates that the changes in cell morphology are due, at least in part, to effects on F-actin organisation, which is regulated by Rho proteins (a member of the Ras superfamily of G-proteins that function as molecular switches) through the formation of lamellipodia and filopodia [30]. Ras hyperactivity is a major factor that drives cell proliferation in a wide range of aggressive neoplasms including CaP. Hyperactivity of members of the superfamily of Ras proteins stems either from overexpression or a mutation-induced loss of the self-regulatory GTPase activity, culminating in constitutive activity [31]. Although mutant Ras is not common in CaP [32], Ras and Ras effectors are chronically activated in advanced CaP [33]. It appears, therefore that PMPMEase activity has a strong effect on the functions of these proteins through its ability to hydrolyse the methylated forms of the proteins. The demethylated forms of the proteins would thus be involved in the promotion of cell proliferation and migration.

EGFR is overexpressed in the form of CaP that pose a major challenge to current treatments [8] as well as in 45% of African-American and 18% of Caucasian CaP cases [34]. Previous studies also revealed high levels of expression of autocrine and paracrine growth factors during CaP progression from localised and androgen-dependent to metastatic and androgen-independent advanced CaP [31]. Increase in activated Ras resulting from activated growth factor receptors leads to advanced CaP [35]. Since signalling by Ras and related proteins occurs downstream of these growth factors and their respective receptors, therapeutic measures that effectively correct hyperactive Ras are likely to be beneficial to patients with cancers that harbour a wider array of upstream dysfunctions involving growth factor receptor hyperactivity. The effects of L-28 inhibition of PMPMEase on cell morphology, F-actin organisation and cell migration is significant from the standpoint of inhibiting metastasis, the process that results in most CaP-related deaths. In addition to the L-28-induced apoptosis observed in the CaP cells, the overexpression of PMPMEase in various CaP tissues implies that it constitutes a target for the development of effective CaP therapies that could block both cell proliferation and metastasis.

The aggressiveness of a tumour is dependent on the ability of the tumour cells to proliferate, invade surrounding tissues, and metastasize to distant tissues [36, 37]. A strong correlation exists between high proliferation rates in metastatic cancers and the overexpression of genes that regulate cell cycle progression, DNA replication, and repair [38]. Treatment of the CaP cell lines with L-28 altered the expression of various cancer-related genes that control these important processes. Cyclin/cyclin-dependent kinases drive cells through the respective cell cycle phases and check points [39]. The frequent deregulation of these enzymes and their regulators in various forms of human cancers is well documented and are therefore considered as therapeutic targets in cancer therapy [40]. Additionally, cyclin-dependant kinase inhibitors also control the activity of the cyclin-dependant kinases, thereby maintaining balance within the cell cycle [41]. In all four CaP cell lines studied, L-28 significantly and concentration-dependently reduced the expression of *CDC2* gene that encodes cyclin-dependant kinase 1 (Cdk1) indicating possible interruption in the cell cycle. Santamaria and coworkers [42] demonstrated that Cdk1 is the most important Cdk when they revealed that in the absence of interphase Cdks, Cdk1 substituted for all the events necessary for cell proliferation to occur.

*MYBL2* expression is common in proliferating cells [43], and its expression is upregulated in metastatic CaP [44]. The down-regulation of this gene by up to 20-fold in the treated cells suggests that the observed L-28 inhibitory effects on cell viability may be attributed to the suppression of cell cycle promotion mediated by Cdk1 and MYB-related protein B. The suppression of BIRC5 (Survivin) gene expression is congruent with the observed effects of L-28 on the cells and anti-apoptotic role of survivin and members of the Ras oncogene family that constitute the putative targets of PMPMEase are known to up-regulate survivin [45]. The suppression of *TOP2A* expression, a marker of cell proliferation in normal and cancerous tissues [46] might imply strand breaks as well as an inability of the treated cells to unwind their DNA for transcription. The growth factor, CTGF has been shown to support angiogenesis and progression of CaP [47]. Also, *CAV1* gene, which encodes for caveolin-1 is upregulated in CaP [48]. Studies have shown a strong correlation between CAV1 up-regulation and increased tumour grade, invasiveness, and regional metastasis and decreased overall survival in the bladder, oesophageal, and CaPs [49]. Epidermal growth factor receptor and its ligands, such as AREG and other paracrine prostate cell growth factors, such as FGF2 [50] were up regulated in the treated cells. These growth factors are known to promote cell proliferation and tumourigenesis and their up-regulation may be a result of the feedback attenuated survival response to the suppressed downstream signalling events. Also, the expression of other important genes for transcription factors such as *E2F1*, *MYB* was reduced in the androgen-dependent cell lines. Cell-protective genes such as *NQO1* were more highly expressed as a consequence of the L-28 treatment.

## Conclusion

The case for anti-PMPMEase targeted therapies to suppress CaP tumour growth is bolstered by the observations in this report that PMPMEase is overexpressed in CaP tissues and cell lines and that its inhibition induces apoptosis in both androgen-dependent and independent CaP cell lines. This implies that the development of more potent polyisoprenylated small molecules to target PMPMEase to effectively manage the more aggressive disease is worthwhile. This is seen not only by the observed inhibition of cell migration, hence the potential anti-metastatic effects, but also the likelihood of inhibition of Ras effects that would help regulate the EGFR hyperactivity seen in all cases of advanced disease.

## Novelty and impact statement

Treatment options for castration-resistant CaP are currently inadequate. This study offers a novel strategy for the development of targeted therapies based on PMPMEase as a biomarker. The data further demonstrate the potential of such agents to mitigate not only the cell proliferation, but also the cell migration that is characteristic of aggressive CaP.

## Conflicts of interest

The authors have no conflicts of interest to declare.

## Figures and Tables

**Table 1.** Cancer-related genes whose levels of expression were significantly altered in androgen-independent prostate cancer cell lines treated with the indicated concentrations of L-28

	Gene name	Accession number	Fold-change			
			DU145 (2 µM)	PC-3 (2 µM)	DU145 (5 µM)	PC-3 (5 µM)
DNA synthesis and repair	*BCL6*	NM_001706.2	2	1	3	2
	*MLL*	NM_005933.2	1	1	2	2
	*MYBL2*	NM_002466.2	-1	-5	-3	-20
	*TYMS*	NM_001071.1	-1	-2	-2	-2
	*TOP2A*	NM_001067.2	-2	-3	-3	-7
	*IRF1*	NM_002198.1	-1	2	2	2
	*MTA1*	NM_004689.2	1	1	2	2
	*MYC*	NM_002467.3	1	1	2	2
	*RRM1*	NM_001033.3	-1	-2	-2	-3
Cell proliferation, growth, and differentiation	*AREG*	NM_001657.2	2	2	6	2
	*BIRC5*	NM_001012270.1	-2	-3	-3	-9
	*IGFBP3*	NM_000598.4	-1	2	-3	2
	*PPARG*	NM_138711.3	2	2	5	2
	*SIAH1*	NM_001006610.1	2	1	2	2
	*EGFR*	NM_005228.3	2	2	4	2
	*IL6*	NM_000600.1	-1	3	2	3
	*TNFRSF10B*	NM_003842.3	2	2	3	2
	*TGFA*	NM_001099691.1	2	2	2	2
	*FGFR3*	NM_022965.2	-1	-2	-2	-3
	*MET*	NM_000245.2	1	2	2	3
	*PIK3CA*	NM_006218.2	1	1	2	2
Cell Cycle regulation and progression	*BIRC5*	NM_001012270.1	-2	-3	-3	-9
	*CCNA2*	NM_001237.2	-1	-2	-2	-4
	*CCND3*	NM_001760.2	-2	-2	-5	-5
	*CCNE1*	NM_001238.1	1	-2	2	-2
	*CDC2*	NM_001786.2	-1	-4	-2	-9
	*CDKN1A*	NM_000389.2	2	2	4	2
	*CDKN2C*	NM_001262.2	-2	-2	-5	-2
	*CEBPA*	NM_004364.2	2	1	2	2
	*MYBL2*	NM_002466.2	-1	-5	-3	-20
	*SIAH1*	NM_001006610.1	2	1	2	2
	*GADD45A*	NM_001924.2	1	2	2	2
Cell migration and adhesion	*CAV1*	NM_001753.3	-4	-1	-7	-2
	*CTGF*	NM_001901.2	-1	-2	-3	-5
	*FGF2*	NM_002006.4	2	2	7	2
	*L1CAM*	NM_024003.2	2	2	2	4
	*LAMB1*	NM_002291.2	1	1	-2	2
	*PLAUR*	NM_002659.2	2	2	4	2
	*SERPINE1*	NM_000602.2	4	2	3	2
Immune and Inflammatory response	*AREG*	NM_001657.2	2	2	6	2
	*IL6*	NM_000600.1	-1	3	2	3
	*MET*	NM_000245.2	1	2	2	3

**Table 2.** Cancer-related genes whose levels of expression were significantly altered in androgen-dependent prostate cancer cell lines treated with the indicated concentrations of L-28

	Gene Name	Accession number	Fold-change			
			22Rv1 (2µM)	LNCaP (2 µM)	22Rv1 (5 µM)	LNCaP (5 µM)
DNA synthesis and repair	*ETS2*	NM_005239.4	1	1	3	3
	*E2F1*	NM_005225.1	-1	-2	-3	-5
	*MYBL2*	NM_002466.2	-2	-2	-4	-3
	*PCNA*	NM_182649.1	-1	-2	-3	-3
	*RAD54L*	NM_003579.2	-1	-3	-2	-4
	*TOP1*	NM_003286.2	1	1	2	2
	*TYMS*	NM_001071.1	-1	-2	-3	-2
Cell proliferation, growth, and differentiation	*BRAF*	NM_004333.3	1	1	3	2
	*EPS8*	NM_004447.5	1	1	2	2
	*ETV1*	NM_004956.3	1	-1	3	-2
	*FGFR1*	NM_015850.2	1	1	7	2
	*IGFBP3*	NM_000598.4	2	1	2	3
	*GRB7*	NM_005310.2	1	-1	3	-2
	*GADD45A*	NM_001924.2	2	1	7	2
	*IFNGR1*	NM_000416.1	1	1	2	2
	*MCL1*	NM_021960.3	1	1	3	2
	*NQO1*	NM_000903.2	2	2	10	4
	*PTPRG*	NM_002841.2	2	-1	4	2
	*PIK3CA*	NM_006218.2	1	1	2	2
	*YES1*	NM_005433.3	1	1	2	2
Cell cycle regulation and progression	*CCNE1*	NM_001238.1	-1	-1	2	-2
	*CDC2*	NM_001786.2	-2	-1	-2	-2
	*E2F1*	NM_005225.1	-1	-2	-3	-5
	*JUN*	NM_002228.3	-1	1	2	2
	*JUN B*	NM_002229.2	1	2	2	3
	*MYB*	NM_005375.2	-1	-1	-2	-2
	*MYBL2*	NM_002466.2	-2	-2	-4	-3
	*PML*	NM_033240.2	1	1	2	2
	*PTEN*	NM_000314.3	2	1	3	2
	*RARA*	NM_000964.2	1	2	2	2
Cell migration and adhesion	*ETV1*	NM_004956.3	1	-1	3	-2
	*JUN*	NM_002228.3	-1	1	2	2
	*TGFBR3*	NM_003243.3	1	-1	3	2
Immune and inflammatory responses	*PTGS2 (COX2)*	NM_000963.1	-2	-2	-2	-3
	*REL*	NM_002908.2	1	1	3	2

**Table 3.** Clinicopathological features of the 114 donors of the prostate tissues used in the TMA study

Characteristics		Patients	
		n	(%)
**Age**	≤65 years	45	39.5
	>65 years	69	60.5
**Normal**		8	7.0
**NAT**		12	10.5
**Histology**	*Adenocarcinoma*	92	80.7
	*Transitional cell carcinoma *	2	1.8
**Grade**	1	9	7.9
	2	37	32.5
	2–3	3	2.6
	3	30	26.3
	4	10	8.8
	Not determined	5	4.4
**Gleason grade**	1	9	7.9
	2	7	6.1
	3	25	21.9
	4	23	20.2
	5	26	22.8
	Not determined	2	1.8
**Gleason score**	1+1	4	3.5
	1+2	1	0.9
	1+3	1	0.9
	1+4	1	0.9
	2+2	8	7.0
	2+3	11	9.6
	2+4	1	0.9
	2+5	1	0.9
	3+2	1	0.9
	3+3	14	12.3
	3+4	6	5.3
	3+5	3	2.6
	4+2	1	0.9
	4+3	5	4.4
	4+4	12	10.5
	4+5	3	2.6
	5+3	2	1.8
	5+4	4	3.5
	5+5	13	11.4

**Table 4.** elationship between PMPMEase staining intensity and clinicopathological features of the prostate cancer tissues used

Characteristics	PMPMEase Staining Intensity, N (%)						*p*-value
	1–100 Trace	101–200 Weak	201–300 Intermediate	301–400 Strong	401–500 Very strong	Mean Scores	
Normal	4 (50.0)	2 (25.0)	3 (25.0)	0	0	162.5 ± 37.50	
NAT	0	3 (25.0)	5 (41.7)	3 (25.0)	1 (8.3)	285.4 ± 23.93	0.0094
**Pathology diagnosis**							
Transitional cell carcinoma	0	0	0	2 (100)	0	391.7 ± 8.3	0.0001*
Adenocarcinomas	0(0)	5(5.4)	17 (18.4)	45(48.9)	25 (27.2)	348.1 ± 8.3	0.0001
**Grade**							
1	0	1(11.1)	0	2(22.2)	6(66.7)	400.0 ± 26.8	0.0001
2	0	1 (2.7)	9 (24.3)	19 (51.4)	8(21.6)	333.4 ± 11.3	0.0001
2-3	0	0	0	3 (100)	0	366.7 ± 15.02	0.0001
3	0	2 (6.7)	6 (20.0)	16 (53.3)	6 (20)	337.9 ± 14.4	0.0001
4	0	1 (10)	2 (20)	4 (40)	3 (30)	335.0 ± 30.2	0.0001
*Undetermined*	0	0	0	0	5 (100)	455.6 ± 12.97	0.0001
**Gleason grade**							
1	0	0	0	4 (44.4)	5(55.6)	410.1 ± 14.10	0.0001
2	0	0	2 (28.6)	3 (42.9)	2 (28.6)	360.7 ± 32.89	0.0001
3	0	1 (4.0)	3 (12)	17 (68.0)	4 (16.0)	343.5 ± 12.42	0.0001
4	0	1 (4.3)	7 (30.4)	10 (45.3)	5 (21.7)	325.6 ± 16.48	0.0001
5	0	3 (11.5)	5 (19.2)	11 (42.3)	7 (26.9)	333.2 ± 17.81	0.0001
*Undetermined*	0	0	0	0	2 (100)	475 ± 25.0	0.0001
**Gleason score**							
1+1	0	0	0	1 (25.0)	3 (75.0)	444.5 ± 26.46	0.0001
1+2	0	0	0	1 (100.0)	0	0	
1+3	0	0	0	1 (100.0)	0	0	
1+4	0	1 (100.0)	0	0	0	0	
2+2	0	1 (12.5)	0	3 (37.5)	4 (50.0)	389.1 ± 28.59	0.0001
2+3	0	0	1 (9.1)	6 (54.5)	4 (36.4)	381.8 ± 15.84	0.0001
2+4	0	0	0	0	1 (100.0)	0	
2+5	0	0	0	0	1 (100.0)	0	
3+2	0	0	1 (100.0)	0	0	0	
3+3	0	0	4 (28.6)	8 (57.1)	2 (14.3	331.3 ± 16.04	0.0001
3+4	0	0	1 (16.7)	4 (66.7)	1 (16.7)	335.4 ± 26.30	0.0043
3+5	0	0	0	2 (66.7)	1(33.3)	362.5 ± 26.02	0.0131
4+2	0	0	1 (100.0)	0	0	0	
4+3	0	0	3 (60.0)	2 (40.0)	0	300 ± 30.87	0.0266
4+4	0	0	4 (33.3)	5 (41.7)	3 (25)	342.7 ± 23.0	0.0001
4+5	0	0	0	3 (100.0)	0	358.3 ± 4.17	0.0129
5+3	0	0	0	2 (100.0)	0	350 ± 12.50	
5+4		1 (25.0)	1 (25.0)	2 (50.0)	0	340.6 ± 58.	0.0243
5+5		2 (15.4)	2 (15.4)	6(46.2)	3 (23.1)	327.9 ± 26.93	0.0001
**Tumour size**							
1	0	0	0	0	2 (100)	451 ± 14.0	
2	0	4 (5.1)	16 (20.3)	40 (50.6)	19 (24.1)	342.2 ± 8.7	0.0001
3	0	0	1 (16.7)	4 (66.7)	1 (16.7)	362.5 ± 26.0	0.0001
4	0	1 (14.3)	0	3 (42.9)	3 (42.9)	383.9 ± 37.7	0.0001

**Table 1.** The effect of L-28 on the viability of normal and prostate cancer cells. The EC_50_ values were obtained from concentration-response curves from 24, 48 and 72 h of exposure to varying concentrations of L-28. Each point represents the mean ± SEM of 4 determinations.

Time (hours)	24	48	72
WPE1-NA22	51.7	30.4	37.7
PC-3	23.0	8.5	4.5
LNCaP	9.4	8.7	4.6
22Rv1	6.8	5.7	1.8
DU 145	70.2	8.2	4.4
